# Intestinal Microbiota of Fattening Pigs Offered Non-Fermented and Fermented Liquid Feed with and without the Supplementation of Non-Fermented Coarse Cereals

**DOI:** 10.3390/microorganisms8050638

**Published:** 2020-04-27

**Authors:** Sebastian Bunte, Richard Grone, Birgit Keller, Christoph Keller, Eric Galvez, Till Strowig, Josef Kamphues, Julia Hankel

**Affiliations:** 1 Institute for Animal Nutrition, University of Veterinary Medicine Hannover, Foundation, Hanover, Bischofsholer Damm 15, 30173 Hanover, Germany; sebastian.bunte@gmx.de (S.B.);; 2 Boehringer Ingelheim Veterinary Research Center GmbH & Co. KG, Bemeroder Straße 31, 30559 Hanover, Germany; 3 Helmholtz Center for Infection Research, Inhoffenstraße 7, 38124 Braunschweig, Germany; 4 Hannover Medical School, Carl-Neuberg-Straße 1, 30625 Hannover, Germany

**Keywords:** gut microbiota, 16S rRNA gene, *Lactobacillaceae*, lactic acid bacteria, probiotics, prebiotics, fermentation

## Abstract

Introducing high numbers of lactic acid bacteria into the gastrointestinal tract of pigs via fermented liquid feed (FLF) could have an impact on intestinal bacterial ecosystems. Twenty piglets were allocated into four groups and fed a botanically identical liquid diet that was offered either non-fermented (twice), fully fermented or partially fermented but supplemented with 40% of non-fermented coarse cereals. Microbiota studies were performed on the small and large intestine digesta and faecal samples. A 16S rRNA gene amplification was performed within the hypervariable region V4 and sequenced with the Illumina MiSeq platform. R (version 3.5.2) was used for the statistical analyses. The digesta of the small intestines of pigs fed FLF were dominated by *Lactobacillaceae* (relative abundance up to 95%). In the colonic contents, the abundance of *Lactobacillaceae* was significantly higher only in the pigs fed the FLF supplemented with non-fermented coarse cereals. Additionally, the digesta of the small and large intestines as well as in the faeces of the pigs fed the FLF supplemented with non-fermented coarse cereals were significantly enriched for two operational taxonomic units (OTUs) belonging to the genus *Lactobacillus* and *Bifidobacterium*. The FLF supplemented with non-fermented coarse cereals had probiotic and prebiotic-like impacts on the intestinal and faecal bacterial composition of pigs.

## 1. Introduction

The fermentation of food and feed for the purpose of preservation has been practiced for hundreds of years in human and animal nutrition [1]. In recent years, the fermentation of liquid feed as a new feeding strategy in pig nutrition has become increasingly widespread. By definition, fermented liquid feed (FLF) is feed mixed with water and fermented for a period long enough to reach steady state conditions, prepared by spontaneous fermentation or inoculation with lactic acid bacteria (controlled fermentation) [2]. The whole feed or only a part (grain fraction) can be fermented, although care has to be taken so that undesirable fermentation does not occur [2]. Van Winsen et al. [3] described the criteria for FLF while studying its effects on bacterial populations of the gastrointestinal tract of pigs. The criteria were as follows: pH values below 4.5, high counts of lactic acid bacteria (> 9 log10 cfu/mL feed), lactic acid concentrations above 150 mmol/L and acetic acid as well as ethanol concentrations below 40 and 0.8 mmol/L, respectively. It is well documented that the main metabolites (i.e., lactic and acetic acid), as well as a low pH, are able to reduce enterobacteria in general [4] and pathogens like *Salmonella* [3]. Introducing high amounts of lactic acid bacteria into the pig’s gastrointestinal tract via FLF, and additionally the supplementation with non-fermented coarse cereals, could have an impact on the intestinal bacterial ecosystem. Previous studies have already shown the influence of FLF on several bacterial species present in the gastrointestinal tract of pigs. To the best of our knowledge, there is no study that has been conducted to examine the possible effects of a supplementation with non-fermented coarse cereals to FLF on intestinal bacterial ecosystems. Increased counts of lactic acid bacteria in the stomach and small intestine contents of pigs and decreased counts of bacteria belonging to the family *Enterobacteriaceae* were commonly observed when offered FLF [3,4,5]. In addition, it has been proven that probiotic lactobacillus strains significantly affect transcriptional patterns within the mucosa in the small intestine that could be correlated to the physiological pathways of metabolic and immune functions [6,7].

Recently, new molecular techniques allow detailed insights into the composition of bacterial ecosystems in comparison with the originally dominating culture-dependent methods [8]. As it is known that gut microbiota plays a critical role in nutrient absorption, metabolism and host immune functions [9], it is of great importance to investigate the possible impacts of current feeding measures on swine gut microbiota. There are researchers outlining that gut microbiota need to be recognized for their capacity to enhance disease resistance, and that the introduction of microbial communities may provide further protection [9]. During the early developmental stages of life, the microbiota is highly sensitive to exogenous perturbations that gains an overall compositional stability with increasing age, which is why newly introduced bacteria barely gain access to the niches in the intestinal tract and fail to become a permanent member of the ecosystem [10]. For example, the findings of Hankel et al. [11] lead to the assumption that a higher age at infection is one of the greatest influencing factors on the load of the enteric zoonotic pathogen *Campylobacter jejuni* within the chicken’s intestinal microbiota.

The aim of the present study was to investigate the intestinal and faecal microbiota of young fattening pigs offered FLF with and without the supplementation of non-fermented coarse cereals. The findings from this investigation might contribute to a better understanding of the possible influences of particular feeding measures on the bacterial ecosystem in the gastrointestinal tract of pigs. With regard to the particular susceptibility of young animals to microbiota perturbations as well as to certain enteric pathogens, the offer of FLF with high contents of lactic acid bacteria could be a chance to establish a bacterial species with beneficial impacts within the intestinal microbiota of young pigs. In addition, the supplementation with non-fermented coarse cereals might stimulate the growth of potentially health-promoting introduced and/or indigenous microorganisms.

## 2. Materials and Methods

A power analysis in R with the pwr package (version 1.3.0) was used to calculate the sample size. The significance level was fixed at the level of 5%, while the power was kept at 80%. To calculate the effect size, values from the literature were taken [3], while counts of lactic acid bacteria in intestinal contents were the primary objective. We considered a minimum difference of two log cfu of lactic acid bacteria between two groups in the contents of the small intestine and one log in the contents of the large intestine as significant. Standard deviation differed about 0.7 to 0.2 log [3]. Therefore, we decided to further calculate with an effect size of 2. The power calculation for two proportions with the same sample sizes and an expected effect size of 2, significance level of 0.05 and a power of 0.8 revealed that four animals should be included into the study. The sample size was adjusted for an expected 10% attrition.

The experiments were carried out in accordance with German regulations. These animal experiments required no notification or approval in accordance with the Animal Protection Act (§ 7, paragraph 2, sentence 3). Interventions before dissection were not carried out. The animals were killed according to § 4, paragraph 3 of the Animal Protection Act, exclusively to use their organs or tissues for scientific purposes. The experiments were approved by the Animal Welfare Officer of the University of Veterinary Medicine Hannover, Germany (“Tötungsanzeigen beim Tierschutzbeauftragten der Stiftung Tierärztliche Hochschule Hannover”, 25.10.2017 and 06.02.2018).

### 2.1. Experimental Design, Animals and Housing

A total of 20 cross-bred piglets (BHZP GmbH, Dahlenburg–Ellringen, Germany; db. Victoria ♀ x db. 77 ♂) were supplied at an age of 61 ± 2 days (mean body weight (BW) of 20.8 ± 2.06 kg) from the Farm for Education and Research in Ruthe (University of Veterinary Medicine Hannover, Ruthe, Germany). The piglets were weaned and vaccinated against *Mycoplasma hyopneumoniae* and Porcine Circovirus 2 (Porcilis PCV M Hyo, Intervet Deutschland GmbH, Unterschleissheim, Germarny) on day 28 of life. The pigs were housed individually in 1 × 3 m boxes with a concrete floor, equipped with a nipple drinker (permanently free access to water) and a manipulable material as enrichment. Visual, olfactory and tactile contact to other pigs was possible at all times. The investigation was conducted in two consecutive experiments (Experiment 1 and Experiment 2). The animals were allocated in four different treatment groups (two groups per experiment with five animals each). The feeding measures differed between each group. In Experiment 1, a non-fermented liquid feed was offered to the control group and compared to the experimental group that was offered FLF. Conversely, in Experiment 2, a non-fermented liquid feed was offered to the control group and compared to the experimental group that was offered FLF supplemented with non-fermented coarse cereals. Both experiments lasted 28 days.

### 2.2. Feeding Regime, Diets and Performance Parameters

The botanical composition of all diets was identical, consisting of the following (in g/kg DM): rye (482 g/kg DM), rapeseed extracted meal (REM, 294 g/kg DM), barley (98 g/kg DM), wheat (98 g/kg DM) and mineral supplement (28 g/kg DM). In Experiment 1 the components of the diets (except REM) were ground by a hammer mill (Rasant–Super^®^, Ley, Sulingen, Germany; diameter of the sieve inset: 3 mm) and mixed with water immediately before feeding, i.e., non-fermented (= common liquid feed, hereinafter referred to as control) or fully fermented for 24 hours prior to its offer. The preparation of the fermented liquid feed was carried out in commercial fermenters (Mini–Fermenter 125 ltr., WEDA, Lutten, Germany). To avoid malfermentation, a freeze-dried, granulated starter culture (Schaumalac Feed Protect XP G, H. Wilhelm Schaumann, Germany), consisting of 1k2079 *Lactobacillus plantarum*, 1k2103 *Pediococcus pentosaceus* and 1k2082 *Lactococcus lactis*, was added at the beginning of each fermentation process in a dosage of 2 × 10^5^ cfu/g liquid feed. After filling, the fermenters were closed immediately and the liquid feeds were stirred therein every hour for 60 s at 900 rpm (semi-anaerob). Equipped with a heating coil, a temperature of 34–38 °C was ensured during the entire fermentation. The FLF was offered to the animals after 24 h of fermentation. After each fermentation process, the corresponding fermenter was thoroughly cleaned and refilled, i.e., fermentation in a batch.

In Experiment 2, the control diet was designed in a similar manner as in Experiment 1. The REM and a part of the rye (ground by a hammer mill) were fermented (almost 60% of the diet) as already described for Experiment 1, while the remaining 40% of the diet, consisting of rye (50%), barley (25%) and wheat (25%), was ground by a roller mill (Haferboy Universal 110^®^, Sommer, Osnabrueck, Germany), but not fermented in order to have varying degrees of comminution (particle size) in the experimental diet (partially fermented). Phytases were added to the control and experimental diet in Experiment 2.

The mineral supplement (as well as the phytase in Experiment 2) was added to the diets after the process of fermentation immediately before feeding. The diets were offered ad libitum fresh twice a day. Each day in the morning, feed refusals were removed before the renewed feed supply. Diets were analyzed by standard procedures in accordance with the official methods of the VDLUFA [12]. The volatile fatty acid content in the liquid diets as well as in digesta were analyzed as already described in [13]. The chemical composition and particle size distribution of the diets are shown in Table 1.

Thermo Scientific™ M.R.S. Agar (de Man, Rogosa, Sharpe, Oxoid Germany GmbH, Wesel, Germany) was used for the selective cultivation and enumeration of lactic acid bacteria. The counts of lactic acid bacteria in the fermented and control diets of Experiment 1 and Experiment 2 are shown in Table 1.

The body weight of the animals was determined at the beginning and at the end of the experiments. The faeces of each animal were collected on day 21 of the experiment and stored immediately at −80 °C (20 samples). Until dissection on day 28 of the experiments, the animals were offered feed. The animals were anaesthetized via an intramuscular injection of ketamine (Ketamidor 100mg/mL^®^, Richter Pharma, Wels, Austria; dosage: 20 mg ketamine/kg body weight (BW)) and azaperone (Stresnil 40mg/mL^®^, Lilly Deutschland, Bad Homburg, Germany; dosage: 2 mg azaperone/kg BW). The anaesthetized animals were euthanized with an intracardial application of T61^®^ (Intervet, Unterschleißheim, Germany; dosage: 0.4 mL/kg BW). Under sterile conditions, the digesta of the small intestine (duodenum) and colon were taken and immediately frozen at −80 °C for further microbiota analyses (40 samples).

### 2.3. 16S rRNA Gene Analyses

A total of 60 samples were stored at −80 °C until the simultaneous analysis. With the help of a mixer mill (Retsch MM 400, Haan, Germany), the chyme was homogenized for 1 min before the DNA extraction was done, based on the DNeasy Blood&Tissue Kit (Qiagen, Hilden, Germany) on an automated liquid handler (Microlab Star, Hamilton Germany GmbH, Gräfelfing, Germany). Before the hypervariable region, the V4 of the 16S rRNA gene was amplified using the primer F515/R806 (in accordance with the previously described protocols [15]) and an additional purification step (Kit: BS 365, BioBasic, Ontario, Canada) was performed. Amplicons were sequenced on the Illumina MiSeq platform (PE250), while the Usearch8.1 software package (http://www.drive5.com/usearch/) was used to assemble, quality control and cluster the obtained reads. –fastq_mergepairs –with fastq_maxdiffs 30 was used to merge the reads. Chimeric sequences were identified and removed with the help of cluster_otus (–otu_radius_pct 3) and the Uchime command included in the Usearch8.1 workflow. Quality filtering was set up with fastq_filter (–fastq_maxee 1); minimum read length was 200 bp before the reads were clustered into 97% ID operational taxonomic units (OTUs). The OTU clusters and representative sequences were determined using the UPARSE algorithm [16]. The taxonomy assignment was done with the help of the Silva database v128 [17] and the Naïve Bayesian Classifier from the Ribosomal Database Project (RDP) [18] with a bootstrap confidence cutoff of 70%.

### 2.4. Statistical Analyses

Statistical analyses of the performance data and fermentation pattern in the digesta were performed using SAS (version 7.1, SAS Institute Inc., Cary, NC, US). The data were analyzed with respect to the factor diet by a one-way analysis of variance (ANOVA) for the independent samples. All statements of statistical significance were based upon *p*-values smaller than 0.05.

Statistical analyses of microbiota were performed using R (version 3.5, www.r–project.org) with the R package “phyloseq” (version 1.24.4) [19]. Due to the addition of phytase to the diets only in Experiment 2, comparisons were made solely between the diets of one experiment. Sample diversity was measured with the species richness estimators Observed Species and Chao 1 indices, whereas the Shannon index characterizes species diversity accounting for the abundance and evenness of the species. Comparisons of sample diversity indices were done using the Wilcoxon rank sum test with *p*-value adjustment method: holm. Ordination was performed on normalized counts using Bray-Curtis dissimilarity-based principal coordinate analysis (PCoA) also provided in the R package “phyloseq”. Factors contributing to the differences in microbial composition of the samples were identified with a permutational multivariate analysis of variance (PERMANOVA) on Bray-Curtis distances. To identify taxa with significantly different abundance, multiple testing also included in the R package “phyloseq” was used on normalized counts. Only the 20 most abundant taxa were included in the multiple testing. *p*-values from this test were adjusted by the Benjamini and Hochberg (“BH”) method to control for the false discovery rate (FDR) of 5%. To find OTUs with significantly different abundance between dietary treatments, counts were compared using the R package DESeq2 (version 1.22.2) which uses tests based on the negative binomial distribution [20]. OTUs were filtered using a false discovery rate (FDR) cutoff of 0.01.

## 3. Results

The general health of each animal was checked at least twice a day. During the experimental phase of four weeks, the animals were healthy. The experiments ran without complications.

### 3.1. Performance Data

The feed intake and feed conversion ratio (FCR = feed requirement in kg per kg body weight gain) were measured individually for the experimental period of four weeks (start on the 62nd day of life). The mean daily feed intake was slightly higher in the groups offered FLF. The FCR was slightly lower in the groups offered FLF. Differences between the groups were not statistically significant (Table 2).

### 3.2. Intestinal Microbiota

The samples with less than 999 total reads were removed. Therefore, only 59 of 60 samples were included in the statistical analyses of the microbiota. The dataset contained 1,495,450 reads (mean number of reads: 25,346; range: 1237–189,600) mapped to 531 OTUs.

#### 3.2.1. Alpha Diversity in Digesta Samples of Small Intestine and Colon

##### Small Intestine

**Figure 1:**
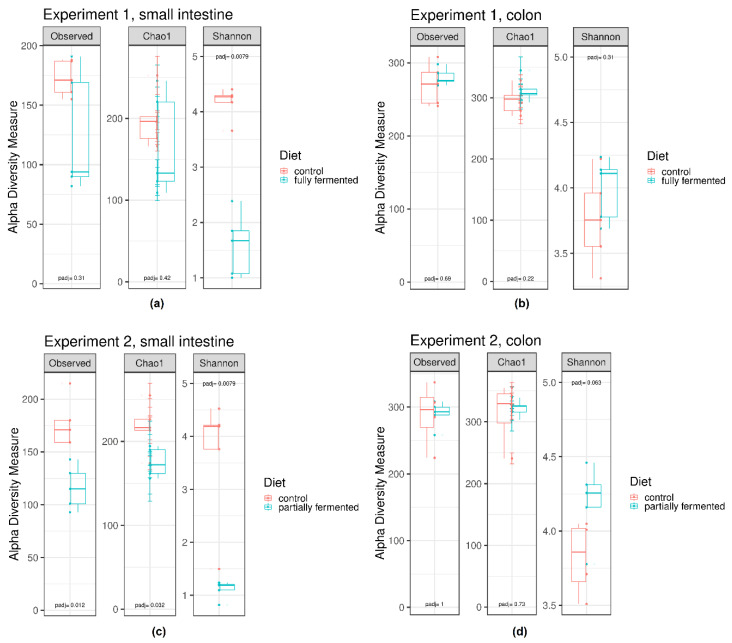
Alpha diversity in intestinal contents depending on the experiment, sampling point and dietary treatment. Box plots showing alpha diversity in samples using the species richness estimators of the Observed Species, Chao1 and Shannon indices. Comparisons of the species richness estimators were done by the Wilcoxon rank sum test with *p*-value adjustment method: holm. (**a**) Experiment 1, small intestine; (**b**) Experiment 1, colon; (**c**) Experiment 2, small intestine; (**d**) Experiment 2, colon.

Comparisons of the Shannon index of samples taken from the small intestine in Experiment 1 revealed significant differences between pigs fed the fully fermented diet and pigs fed the non-fermented control diet, whereby differences in the Observed Species and Chao 1 indices were not significant between the dietary treatments. Conversely, for Experiment 2, differences of all the measured species richness estimators, such as the Observed Species, Chao 1 and Shannon indices, in the samples taken from the small intestine were significant between dietary treatments. Alpha diversity in the samples taken from the small intestine of the animals offered the fermented diet, regardless of whether the diet was fully or partially fermented, was lower compared with the animals fed the non-fermented control diet (Figure 1A,C). The Shannon index, in particular, showed the greatest differences between the dietary treatments. Further, while the Observed Species and Chao 1 indices are species richness estimators based on abundance (estimate the number of species in a community, does not consider the number of individuals of each species), the Shannon index characterizes species diversity accounting for the abundance and evenness of the species. This indicates that after the offer of fermented liquid diets, the intestinal contents of the small intestine hosted less varied bacterial species with less even distribution. Those species present had a higher relative abundance.

##### Colon

Comparisons of the measured species richness estimators—the Observed Species, Chao 1 and Shannon indices—in the samples of the colonic contents of the animals fed different diets revealed no statistically significant differences (Figure 1B,D). Even though it was not statistically significant, the Shannon index in the samples of the animals fed a fermented diet was numerically higher compared with the animals offered the non-fermented control diet. The difference in the Shannon indices between the samples of the animals fed the control and the partially fermented liquid feed was greater (padj = 0.063) than the difference between the samples of the animals fed the control and the fully fermented liquid feed in Experiment 1 (padj = 0.31). This indicates that after the offer of fermented diets, the intestinal contents of the colon hosted a similar number of different bacterial species but the individuals among the bacterial species seemed to be more evenly distributed.

#### 3.2.2. Alpha and Beta Diversity in Faecal Samples

**Figure 2:**
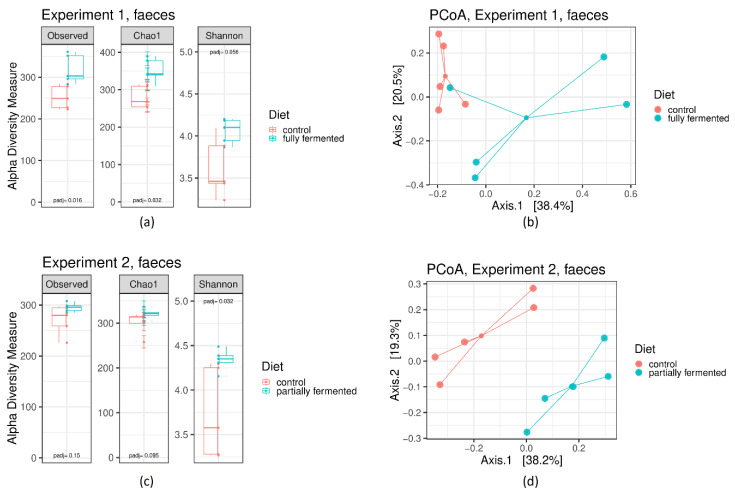
Alpha diversity in the faecal samples (day 21 of the experiment) depending on experiment and dietary treatment. Box plots showing alpha diversity in samples using the species richness estimators of the Observed Species, Chao1 and Shannon indices. Comparisons of the species richness estimators were done by the Wilcoxon rank sum test with *p*-value adjustment method: holm. A Bray-Curtis dissimilarity-based principal coordinate analysis (PCoA) was performed on the faecal samples. Each point represents a different animal; colored lines connect animals of one dietary treatment (control, fully fermented or partially fermented): (**a**) Alpha diversity in Experiment 1; (**b**) PCoA in Experiment 1; (**c**) Alpha diversity in Experiment 2; in (**d**) PCoA in Experiment 2.

Comparisons of the measured species richness estimators in the faecal samples of the animals in both experiments revealed less species richness in the groups fed the control diet compared with the animals fed the fermented feed (Figure 2). Except for one animal in Experiment 1, the principal coordinate analysis ordination of a Bray-Curtis dissimilarity matrix (PCoA, Figure 2) revealed that the samples of the animals belonging to an identical dietary treatment are ordinated closer compared with the animals offered a different diet, which shows a higher similarity of faecal microbiota between the animals within one group and a higher dissimilarity between the groups. This demonstrates that the influences of the feeding measures on microbiota are still present in the pig’s faeces. Permutational multivariate ANOVA, with Bray-Curtis dissimilarities, revealed the diet as the factor that contributes significantly to the differences in the microbial composition of the faecal samples, explaining 23.7% of the sample’s variability (*p* = 0.001).

#### 3.2.3. Relative Abundance per Sample and Effects of the Diet on the Phylum, Family and OTU Level

##### Small Intestine

**Figure 3:**
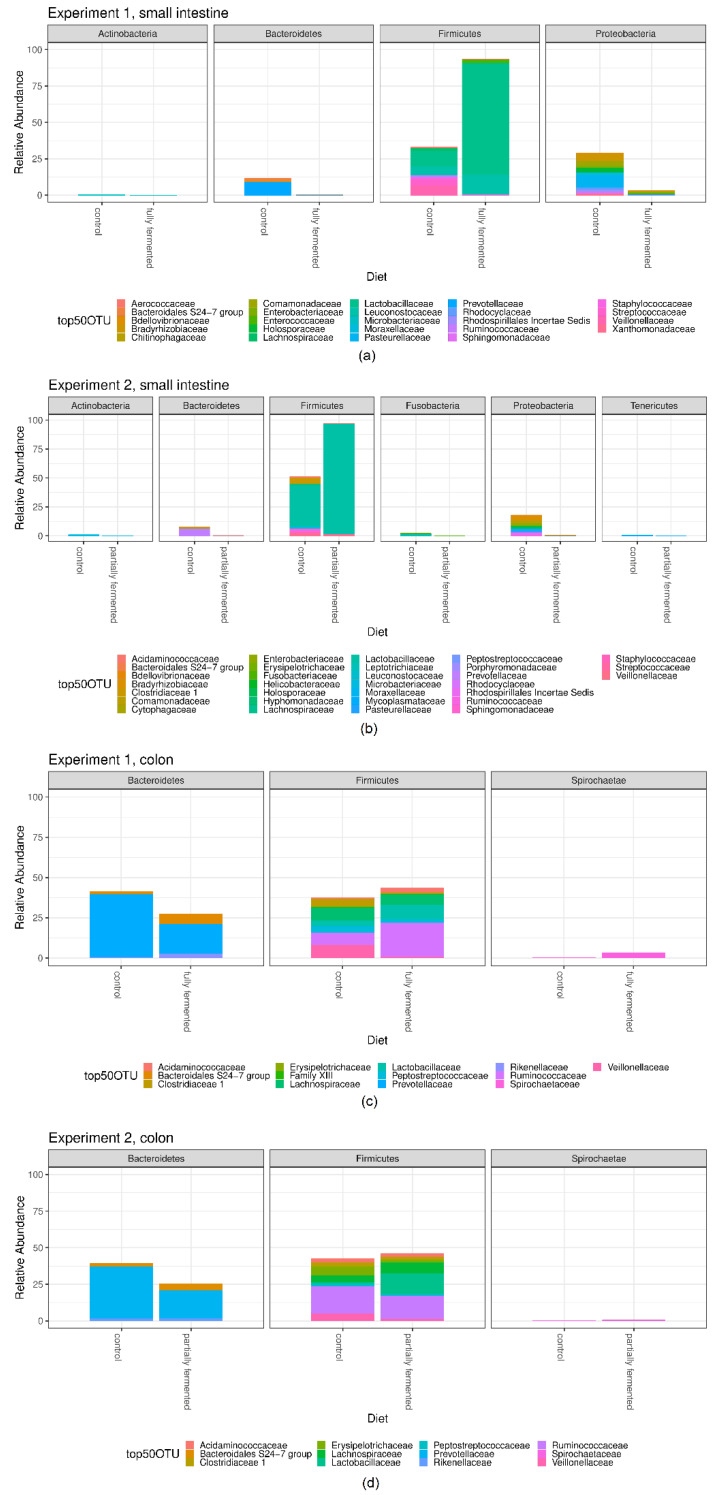
Relative abundance of the 50 most abundant operational taxonomic units (OTUs) belonging to the bacterial families within the different phyla in (**a**) Experiment 1, small intestine; (**b**) Experiment 2, small intestine; (**c**) Experiment 1, colon, and (**d**) Experiment 2, colon.

Independent of the dietary treatment, the microbiota of the small intestine was dominated at the phylum level by *Firmicutes* and *Proteobacteria*, followed by *Bacteroidetes*. If a fermented diet was used instead of a non-fermented diet, the proportion of *Firmicutes* increased (from 44.0% to 94.7% in Experiment 1; from 59.5% to 97.9% in Experiment 2), while the proportion of *Proteobacteria* decreased, even below 1% relative abundance in Experiment 2 (Figure 3, Table 3).

Multiple testing on the normalized counts on each phylum yielded a significantly different count profile between the dietary treatments in both experiments for *Firmicutes*, *Proteobacteria* and *Bacteroidetes* (Experiment 1: adjusted *p* = 0.00794; Experiment 2: adjusted *p* = 0.01058).

Multiple testing on the normalized counts yielded 7 (of 11) families that showed a significantly different count profile between the dietary treatments (Supplementary Table S1). The strongest difference between the groups was seen between *Lactobacillaceae*, *Bradyrhizobiaceae*, *Streptococcaceae*, *Holosporaceae*, *Veillonellaceae*, *Rhodospirillales Incertae Sedis* and *Prevotellaceae* (adjusted *p* = 0.01247). In Experiment 2, multiple testing on the normalized counts yielded 9 (of 15) families that showed a significantly different count profile between the dietary treatments (Supplementary Table S2), with strongest differences between *Lactobacillaceae*, *Comamondaceae*, *Bradyrhizobiaceae*, *Rhodospirillales Incertae Sedis*, *Holosporaceae*, *Leuconostocaceae*, *Ruminococcaceae*, *Prevotellaceae* and *Lachnospiraceae* (adjusted *p* = 0.01323). The relative abundance of *Lactobacillaceae* reached its highest values in Experiment 2 when offered a partially fermented diet (95%, Table 4).

**Figure 4:**
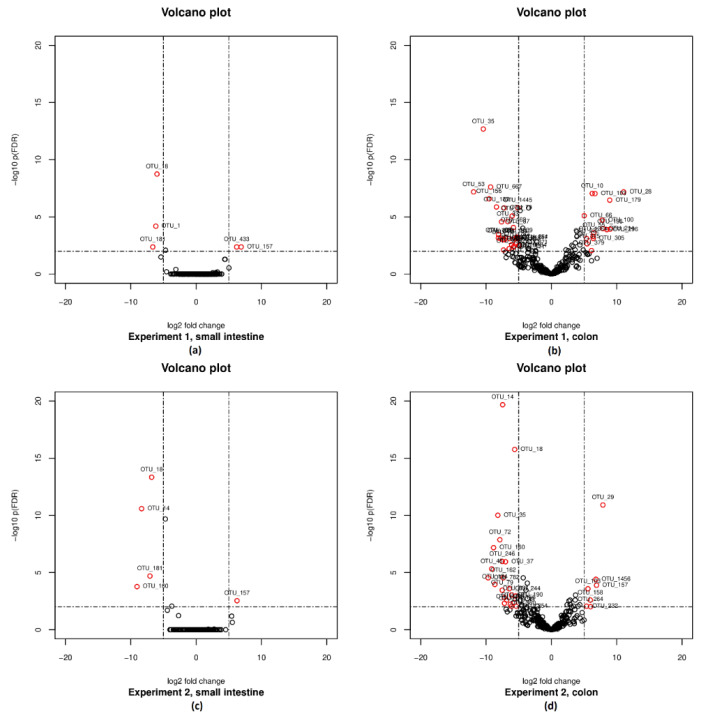
Volcano plot visualizing –log10 false discovery rate (FDR)-adjusted *p*-values versus log2 fold changes for all 531 OTUs. The horizontal lines show the significance threshold for an FDR < 0.01. Each open point represents a single OTU in (**a**) Experiment 1, small intestine; (**b**) Experiment 1, colon; (**c**) Experiment 2, small intestine, and (**d**) Experiment 2, colon. Open points above the significance threshold and beyond the log2 fold change criterion of ±5 are colored red.

At the species level, 6 (Experiment 1) to 7 (Experiment 2) of 531 OTUs showed a significantly (plus an additional log fold change criterion of ± 5) different abundance between the dietary treatments, where 1 (Experiment 2) to 2 (Experiment 1) of these OTUs were more enriched in the animals offered the non-fermented control diet. Four OTUs were more enriched in the animals offered the fully fermented diet in Experiment 1 and six more enriched in the animals offered the partially fermented diet in Experiment 2. The log fold changes for these OTUs are shown in the Supplementary Materials’ Table S2 and plotted in Figure 4. In both experiments, OTU_18 had the smallest *p*-value, followed by OTU_1 and OTU_181 in Experiment 1, and OTU_14, OTU_72, OTU_181 in Experiment 2, all representing the bacterial species that are assigned to the genus *Lactobacillus*, which belongs to the family *Lactobacillaceae*. All of them were enriched in the animals’ digesta which were fed the fermented diets. On the other hand, in both experiments, the OTU_157 was enriched in the animals offered the non-fermented control diet. OTU_157 represents a bacterial species that is assigned to the genus *Leuconostoc*, which belongs to the family *Leuconostocaceae* within the order *Lactobacillales* as well. The relative abundance of the genus *Leuconostoc*, which is mainly formed by OTU_157, amounted in the animals of Experiment 1 offered the non-fermented control diet to 0.371% (fully fermented: 0.033%) and to 0.888% in Experiment 2 (partially fermented: 0.003%).

##### Colon

The microbiota of the colon was dominated at the phylum level by *Firmicutes* and *Bacteroidetes* (Figure 3, Table 1). Multiple testing on the normalized counts on each phylum did not yield any phyla that showed a significantly different count profile between the dietary treatments, neither in Experiment 1 nor in Experiment 2.

After merging the 20 most abundant OTUs to the taxonomic level family, multiple testing on the normalized counts yielded 3 (of 9) families in Experiment 2 that showed a significantly different count profile between the dietary treatments (Supplementary Table S1), whereas in Experiment 1, no family showed a significantly different count profile. The strongest differences between the groups in Experiment 2 were seen between *Veillonellaceae*, *Lactobacillaceae* and *Erysipelotrichaceae* (adjusted *p* = 0.04762).

At the species level, 69 of 531 OTUs showed a significantly (plus an additional log fold change criterion of ± 5) different abundance between the dietary groups in Experiment 1, where 27 of these OTUs were more enriched in the animals offered the non-fermented control diet and 42 were more enriched in the animals offered the fully fermented diet. The log fold changes for these 69 OTUs are shown in the Supplementary Materials’ Table S2 and plotted in Figure 4. OTU_35 had the smallest *p*-value. Neither OTU_18 nor OTU_1, which were already tested as significantly different in the same animals but at a different location in the small intestine, could be retrieved among the significantly different abundant OTUs. However, OTU_181 could be found again under the significant different OTUs in the colon.

At the species level, 47 of 531 OTUs showed a significantly (plus an additional log fold change criterion of ± 5) different abundance between the dietary groups in Experiment 2, where 17 of these OTUs were more enriched in the animals offered the non-fermented control diet and 30 were more enriched in the animals offered the partially fermented diet. The log fold changes for these 47 OTUs are shown in the Supplementary Materials’ Table S2 and plotted in Figure 4. OTU_14 had the smallest *p*-value, followed by OTU_18, both representing the bacterial species that are assigned to the family *Lactobacillaceae*. Besides, OTU_18 and OTU_14, OTU_72 and OTU_160 could be retrieved among the significantly different abundant OTUs in the colon as well as in the samples of the small intestine. In Experiment 2, the relative abundance of *Lactobacillaceae* in the colonic contents amounted to 14.2% when offered a partially fermented diet (control diet: <1%).

Similar numbers of the genus *Leuconostoc* found in the small intestine of the animals fed the control diets seemed to be transferred to the colon only in Experiment 2 (0.063% in the colonic contents of Experiment 1, 0.809% in the colonic contents of Experiment 2). Therefore, the abundance of OTU_157 differed significantly (plus an additional log fold change criterion of ± 5) between the dietary groups only in Experiment 2.

##### Faeces

In Experiment 2, OTU_18 and OTU_160 could be found continuously among the significantly different abundant OTUs in the samples of the small intestine, the colon and finally in the faecal samples as well (Supplementary Materials’ Table S2). In the course of the entire intestinal tract, OTU_18 and OTU_160 were always enriched in the samples of the animals fed the partially fermented diet. OTU_160 represents a bacterial species that is assigned to the genus *Bifidobacterium,* which belongs to the family *Bifidobacteriaceae*. The relative abundances of *Bifidobacteriaceae* in the intestinal samples are shown in Table 4.

### 3.3. Fermentation Pattern in Digesta of Small Intestine and Colon

Significant differences between the dietary treatments were found with regard to l- and d-lactate. The l-lactate concentration in the digesta of the small intestine is about 3 (Experiment 1) to 10 times (Experiment 2) higher, while the d-lactate was even 17 (Experiment 2) to 37 (Experiment 1) times higher, when offered the fermented feed. The differences in the bacterial fermentation products n-butyrate, acetate and propionate between the dietary treatments within one experiment were not significant, neither in the digesta of the small intestine nor in the digesta of the colon (Table 5).

When the concentrations of the measured volatile fatty acids in the colonic contents of the control groups are set to 100%, the following deviations of the experimental groups from the control groups occur: 97.2% of acetate, 92.3% of propionate and 125% of n-butyrate in Experiment 1, and 113% of acetate, 117% of propionate and 134% of n-butyrate in Experiment 2. The differences in the concentrations of the volatile fatty acids in the colonic contents were greater between the pigs fed the partially fermented feed supplemented with non-fermented coarse cereals and the pigs fed the non-fermented liquid feed in Experiment 2 compared with the differences between the groups in Experiment 1.

## 4. Discussion

The particular susceptibility of young animals to microbiota perturbations possibly represents an increased risk of disease, but also might give the opportunity to establish bacterial species with beneficial impacts. Diet can be a major driver of population dynamics of the small intestine microbiota [21]. The offered FLF is characterized by pH values below 4.0, high counts of lactic acid bacteria (above 9 log10 cfu/g diet) and high amounts of lactic acid. Those characteristics have an impact on the intestinal bacterial ecosystem of young fattening pigs.

The FLF in the current study showed a two-point lower pH compared with the non-fermented control diet. Furthermore, the pH of the contents taken from the small intestine were significantly lower in the groups fed the FLF compared with the groups fed the non-fermented control diet. In an ex vivo trial, Koop [22] evaluated the specific influence of the stomach content which originated from the gastric fundus region of weaned pigs on the survival of *Salmonella* Derby. A different physical form of the diet had an impact on the pH and simultaneously on *S*. Derby counts in the stomach contents. Further, while a multiplication of this pathogen was seen in the stomach contents with a pH of about 5.09, a rapid reduction of *S*. Derby was observed in the stomach contents with a lower pH (about 2.48). In addition, a significantly higher content of lactic acid in the contents of the small intestines were found in the animals fed the FLF in the present study. Lactic acid inhibition of *Salmonella* Typhimurium is related to the present pH [23]. Lactic acid enters the bacterial cell in the undissociated state and dissociates inside when the intracellular pH is higher compared with the external pH. The dissociated moiety accumulates because lactic acid could not leave the cell in this form, consequently lowering the pH and inhibiting *S*. Typhimurium [23]. If the stomach acts as the first line of defense against certain pathogens, it can be supposed that under the present feeding measures, the small intestine might represent a second line of defense.

This second line of defense might benefit from a further contributing factor, the so-called “competitive exclusion”. The competitive exclusion principle states that complete competitors cannot coexist at constant population values and the one with the slightest advantage will dominate in the long term, while the other one will become extinct [24]. This concept was already used in the 1970s to combat pathogens with zoonotic relevance like *Salmonella* by administering the gut contents of adult chickens to young chicks [25]. Nowadays, faecal microbiota transplantation has been effective in treating recurrent *Clostridium difficile* infection with success in humans [26], but it has to be cautiously noted that bacteria may not be the only player in donor faeces that affects the recipient’s biology [27] and that faecal transplants might cause health and safety problems to animals, while some associations of lactic microflora could be suitable as competitive exclusion against intestinal pathogens [28]. Zacconi, Scolari, Vescovo and Sarra [28] evaluated the competitive exclusion activity of fermented milk (kefir) high in yeasts and lactic acid bacteria on *Campylobacter jejuni* colonization in chickens, showing a reduction of *C. jejuni* counts in kefir-treated chicks up to 6–7 log CFU/g caecal content. The authors suspect the reasons for the results are in the complexity of the microflora association in the used fermented product. Many of the species found in fermented food are phylogenetically related to probiotic strains, and can therefore be a dietary source of living microorganisms that may contribute to human health in a similar manner stated for probiotics [29]. To behave as a probiotic, lactic acid bacteria must be able to survive passage through the upper gastrointestinal tract and the doses should reflect any losses inherent to the probiotic as it moves through the gastrointestinal tract [30]. Lactobacilli counts found in the gastrointestinal contents of 48-days old pigs decrease clearly (about one log level) after the passage through the stomach [31]. Even if they are formulation- and strain-dependent, studies with positive effects that can be traced to the probiotic use are rare when using counts below 10^8^ cfu per day, and levels much higher may be needed for certain effects [30]. Therefore, increasing the levels of live cultures may be of interest to those wishing to take full advantage of the benefits [30]. Microbial density increases along the intestinal tract. The total cultivable bacteria in the small intestine contents of 48-days old pigs amounted between 8 and 9 log cfu/g digesta, while in the colon about 10 log cfu/g digesta could be found [31]. With the offer of the FLF in the present study, the animals ingested about 2 to 4 × 10^12^ cfu of lactic acid bacteria per day, 10,000 times more compared with the stated minimal dose for probiotics by Douglas et al. [30]. Therefore, it is not surprising that the introduced amount of lactic acid bacteria flooded the small intestine of pigs in the present study. This was shown in the present study by the measured alpha diversity indices, especially the Shannon index, which were significantly lower in the pigs offered FLF. Further, while achieving a similar microbe domination in the densely colonized large intestine seems barley feasible, overcrowding the microbiota in the small intestine is feasible [21]. It can be assumed that the presence of the high amount of the introduced lactic acid bacteria via FLF could have suppressed the abundance of commonly occurring lactic acid bacteria. OTU_157 represents a lactic acid bacterium that was significantly enriched in the animals offered the non-fermented control diets in both experiments. This lactic acid bacterium seems to be a commonly occurring bacterium in the intestinal flora under the conditions of keeping and feeding in the present experiments. In the small intestine, the principle of competitive exclusion seems to have been successfully established with the administration of FLF and further studies to test the feeding measures for a possible increased resistance towards intestinal pathogens are of special interest.

It has to be underlined that the species richness in the colon digesta expressed by the Observed Species and Chao 1 indices were similar between the groups, while the Shannon index revealed even numerically higher values in the groups of both experiments fed the FLF, which indicates that the individuals among the bacterial species seem to be more evenly distributed. Further, alpha diversity in faecal samples was always numerically higher in groups fed the FLF and the differences between the dietary treatments within the experiments of at least one of the measured alpha diversity indices were significant. The loss of intestinal microbiota diversity appears as the most constant finding of dysbiosis or an unbalanced microbiota and is associated with certain human diseases [32]. For this reason, these effects on the microbiota of the large intestine should generally be considered beneficial.

Even though they did not dominate the bacterial ecosystem in the colon, a higher abundance of lactic acid bacteria was found in the colon as well. However, the differences in the relative abundance of the family *Lactobacillaceae* in the colonic contents were only significant between the dietary treatments in Experiment 2. Three of the OTUs in Experiment 2 belonging to the genus *Lactobacillus* were enriched in the small and large intestine digesta of the pigs fed FLF. Moreover, one of those *Lactobacilli* was found in the faeces with significant differences between the feeding groups being enriched in the animals fed FLF supplemented with non-fermented coarse cereals. Similar results were found for one OTU that could be assigned to the genus *Bifidobacterium*. Those observations were limited to Experiment 2 as well. About 25.5% of the particles in the partially fermented diet supplemented with non-fermented coarse cereals were greater than 2 mm. It can be assumed that due to the supplementation of non-fermented coarse cereals to the FLF in Experiment 2, a higher amount of carbohydrates escaped digestion and absorption in the small intestine and reached the large intestine whereby it became available as a substrate for the present bacterial flora. The assumption can be supported by the observation of the single intact rolled cereals in the faeces of the animals, which only occurred when the partially fermented diet was offered. The starch content in the faeces of the animals offered the partially fermented diet supplemented with non-fermented coarse cereals amounted to 129 g/kg DM, being four times higher compared with the animals fed the fully fermented diet and still almost three times higher compared with the faeces of the animals fed the non-fermented liquid feed in Experiment 2. Therefore, even if not measured, still higher starch contents can be suspected in the colon. This could have favored the growth of specific bacterial species. For this reason, it was expected that more substrate would lead to more bacterial products: e.g., volatile fatty acids. The concentrations of all measured volatile fatty acids (acetic acid, butyric acid and propionic acid) were higher in the colonic contents of the pigs fed the partially fermented feed supplemented with non-fermented coarse cereals compared with the pigs fed the non-fermented liquid feed. Further, in the colonic contents of the pigs in Experiment 1 fed the fully fermented diet, the measured volatile fatty acids were slightly lower (except butyric acid) compared with the pigs fed the non-fermented control diet. For this reason, an additionally prebiotic-like effect can be assumed. Compared with a probiotic, which pursues the goal of introducing exogenous bacteria into the colonic microbiota, prebiotics should stimulate the growth of one or a limited number of potentially health-promoting indigenous microorganisms [33].

It can be concluded that the offer of FLF had an influence on the bacterial composition in the small intestine, large intestine and up to faeces. With the supplementation of non-fermented coarse cereals to FLF, additional prebiotic-like effects were encouraged. For the future, infection studies with the aim of increasing the resistance of animals to intestinal pathogens through similar feeding measures can be of special interest. This interest might go beyond animal health and could also be important with regard to pathogenic germs with zoonotic potential. Finally, as pigs are the preferred model for research into dietary modulation of the human gut microbiota [34], these findings could be of interest not only for animal nutrition but also in the field of human nutrition as well.

## Figures and Tables

**Table 1:** Chemical composition (g/kg DM) of the diets, lactic acid bacteria counts (log10 cfu/g diet) as well as proportion of particle sizes (% of DM) measured by wet sieve analysis.

	Experiment 1		Experiment 2	
Diet	Control	Fully Fermented	Control	Partially Fermented
DM (g/kg as fed)	213	213	216	216
Crude ash	48.5	49.5	47.8	50.4
Crude protein	199	201	195	195
Crude fat	28.2	25.3	28.3	27.1
Crude fibre	55.9	59.2	60.2	59.1
Starch	422	425	452	459
Sugar	71.2	18.4	68.4	27.3
L-lactate	0.103	26.2	0.104	16.7
D-lactate	0.052	27.5	0	15.5
Acetic acid	0.723	8.28	0.862	6.65
Butyric acid	0.012	0.013	0.019	0.009
pH	5.95	3.75	5.95	3.95
Lactic acid bacteria	4.91	9.31	4.96	9.05
Particle size				
≥2 mm	7.28	1.59	6.37	25.5
≥1–<2 mm	32.0	11.6	30.6	14.8
≥0.2 mm–<1 mm	28.0	21.3	27.0	17.8
<0.2 mm	32.8	65.5	36.1	41.9
GMD ^1^	476	203	439	470

^1^ GMD: Geometric Mean Diameter [14].

**Table 2:** Performance parameters of pigs depending on feeding concept.

	Experiment 1		Experiment 2	
Performance Parameters	Control	Fully Fermented	Control	Partially Fermented
Mean daily feed intake (g)	1932 ± 212	1964 ± 196	1892 ± 79.3	1922 ± 219
Final body weight (kg) ^1^	47.0 ± 4.43	47.3 ± 3.78	49.4 ± 2.39	48.6 ± 3.49
FCR (kg/kg)	1.97 ± 0.091	1.99 ± 0.108	1.98 ± 0.082	2.09 ± 0.104

^1^ at day 28 of the experiments (age of the pigs: 90 days).

**Table 3:** Relative abundance (%) of bacterial phyla depending on the dietary treatment and sampling point in Experiment 1 and Experiment 2.

Experiment 1	Small Intestine		Colon	
Phylum	Control	Fully Fermented	Control	Fully Fermented
*Firmicutes*	44.0	94.7	51.7	58.1
*Proteobacteria*	34.5	3.66	0.717	0.789
*Bacteroidetes*	15.9	1.11	34.8	46.1
*Actinobacteria*	2.87	0.214	0.547	0.373
*Spirochaetae*	0.274	0.050	0.400	5.28
**Experiment 2**	**Small Intestine**		**Colon**	
**Phylum**	**Control**	**Partially Fermented**	**Control**	**Partially Fermented**
*Firmicutes*	59.5	97.9	54.6	63.6
*Proteobacteria*	21.8	0.91	0.616	0.610
*Bacteroidetes*	11.9	0.680	43.8	32.3
*Actinobacteria*	1.73	0.338	0.253	1.02
*Fusobacteria*	2.69	0	<0.01	0
*Spirochaetae*	0.443	0.020	0.384	1.86

**Table 4:** Relative abundance (%) of the bacterial families *Bifidobacteriaceae* and *Lactobacillaceae* depending on the dietary treatment and sampling point in Experiment 1 and Experiment 2.

Experiment 1	Small Intestine		Colon	
Family	Control	Fully Fermented	Control	Fully Fermented
*Bifidobacteriaceae*	0	0.002	0.101	0.001
*Lactobacillaceae*	10.7	76.4	3.46	9.22
**Experiment 2**	**Small Intestine**		**Colon**	
**Family**	**Control**	**Partially Fermented**	**Control**	**Partially Fermented**
*Bifidobacteriaceae*	0	0.253	0.001	0.601
*Lactobacillaceae*	35.2	95.0	0.776	14.2

**Table 5:** Means of dry matter (DM, in g/kg fresh matter), l-lactate, d-lactate, volatile fatty acids (in g/kg DM) and pH in the digesta of the small intestine and colon depending on the dietary treatment.

	Experiment 1		Experiment 2	
Small Intestine	Control	Fully Fermented	Control	Partially Fermented
DM	143	115	156	130
L-lactate	11.8 ^b^	40.3 ^a^	3.62 ^b^	38.6 ^a^
D-lactate	0.429 ^b^	16.1 ^a^	1.06 ^b^	18.4 ^a^
Acetic acid	6.11 ^a^	5.14 ^b^	3.57 ^a,b^	2.37 ^b^
Propionic acid	0.093 ^b^	0.088 ^b^	0.161 ^a,b^	0.235 ^a^
Butyric acid	ND	0.007	0.142	0.037
pH	6.61 ^a^	5.73 ^b^	6.59 ^a^	6.14 ^c^
**Colon**				
DM	224	193	207	199
L-lactate	0.035	0.016	0.020	0.084
D-lactate	0.081	0.049	0.069	0.075
Acetic acid	25.4	24.7	31.3	35.3
Propionic acid	16.9 ^b^	15.6 ^b^	22.7 ^a,b^	26.6 ^a^
Butyric acid	10.2	12.7	10.8	14.5
pH	5.79 ^b^	6.26 ^a^	5.81 ^b^	5.74 ^b^

ND, not detectable, value below detection limit; ^a,b,c^ values within one row with different superscripts differ significantly at *p* < 0.05.

## Supplementary Materials

The following are available online at https://www.mdpi.com/2076-2607/8/5/638/s1. Table S1: Comparison of bacterial relative abundance in the small intestine and colonic contents (at taxonomic level, family) between the dietary treatments, Table S2: OTUs with significant (plus an additional log fold change criterion of ±5) different abundance between dietary treatments in Experiment 1 and Experiment 2.
